# A Green Enzymatic Extraction Optimization and Oxidative Stability of Krill Oil from *Euphausia Superba*

**DOI:** 10.3390/md18020082

**Published:** 2020-01-27

**Authors:** Li Zhou, Fu Yang, Minghao Zhang, Jikai Liu

**Affiliations:** 1 School of Pharmaceutical Sciences, South-Central University for Nationalities, Wuhan 430074, China; yangfu19951112@icloud.com (F.Y.); minghaozhang@scuec.edu.cn (M.Z.); 2 College of Food Science and Technology, Nanjing Agricultural University, Nanjing 210023, China

**Keywords:** carboxymethyl chitosan, krill oil, oxidative stability, controlled release, nanoliposome

## Abstract

Krill oil enriched with polyunsaturated fatty acids is in the form of phospholipid. However, its application as a dietary supplement is limited, because of its rapid deterioration. Thus, this study aims to investigate the oxidative stability of krill oil extracted from *Euphausia superba*. Under optimal conditions (enzyme concentration 0.16%, enzymolysis time 2.9 h, and enzymolysis temperature of 45 °C) designed by response surface methodology, the extraction yield of krill oil is 86.02%. Five assays, including peroxide value (POV), thiobarbituric acid-reactive substances (TBARS), pH value, and turbidity were used to determine the oxidative stability of krill oil nanoliposomes during storage. Carboxymethyl chitosan (CMCS) nanoliposomes showed a significant reduction in POV and TBARS values, a prevention of pH value decrease and turbidity increase. This study indicated that CMCS nanoliposome can effectively improve the oxidative stability of krill oil during storage. Furthermore, the release profile in vitro illustrated that the controlled release of krill oil carried out by CMCS nanoliposomes is feasible.

## 1. Introduction

Omega-3 polyunsaturated fatty acids (omega-3 PUFAs) have many functions for human health, including the prevention of cardiovascular diseases, and a reduction in the risks of inflammation, cancers, and renal disorders. Hence, an adequate intake of omega-3 PUFA is important. Supplementation of food formulations with omega-3 PUFA has recently emerged as an interesting method of food consumption. Fish oil is an important source of omega-3 PUFA that is widely used in dietary supplements [1,2]. However, recent studies suggested that not all omega-3 PUFA are equal. The natural molecular forms of omega-3 PUFAs are typically triglycerides (fish oil) and phospholipids (krill oil). The phospholipid omega-3 PUFA of krill oil is proven to be more bioavailable compared with fish oil [3,4,5]. Therefore, krill oil has recently been considered as an alternative source of omega-3 PUFA oils.

Phospholipids are functional components in food. They play important roles in human health, especially in protecting the cardiovascular system and in improving memory and learning. Thus, the extraction methods that enable the determination of the correct content of phospholipids in foods are highly important. An incomplete extraction of total lipids would cause serious errors in phospholipid quantification. The Bligh and Dyer method and Folch method are traditional methods to extract the total lipids from various food matrices [2,6,7]. However, these methods need high amounts of organic solvents and most of them are hazardous. Enzymatic methods that are in line with the concept of green chemistry have many advantages, such as being non-toxic, mild treatment conditions, having a high specificity, and fewer by-products. However, few studies have investigated the optimal parameters of enzymatic hydrolysis for the extraction of krill oil from *Euphausia superba*. The selection of suitable enzymes is closely linked with the composition, proportion and complexity of the cell wall structure of marine organisms [8]. Furthermore, the optimization of exaction conditions of enzymatic hydrolysis are important for lipid recovery. For example, inappropriate temperature, enzyme concentration, and time could decrease the enzyme activity, and even lead to protein denaturation. Thus, we report here the optimization of the enzymatic hydrolysis extraction of krill oil from *Euphausia superba* using the response surface methodology (RSM) based on a Box–Behnken design (BBD). The primary process variables affecting the extraction yields include enzyme concentration, enzymolysis time and temperature.

Krill oil contains approximately 35–93% phospholipids and 12–30% triglycerides, with a small quantity of diacylglycerols, cholesterol, and free fatty acids [9]. The inherent combination of surface-active lipids, such as phospholipids, diacylglycerols, and free fatty acids, as well as triglyceride, could promote the formation of oil-in-water (O/W) emulsions, without the help of additional emulsifiers, thus allowing for supplementation in food [10]. However, one of the major problems associated with krill oil is its high susceptibility to oxidative deterioration. Lipid oxidation leads to the development of undesirable off-flavors and potentially toxic compounds, and a decline in the nutritional value of oils. Thus, it is necessary to prevent krill oil from being oxidized during food processing or during their transport to the required target site in the human body. The solution may lie in the encapsulation of krill oil in order to improve the oxidative stability. Nanoparticles are effective delivery systems for preventing functional components from oxidative decomposition.

Chitosan is a cationic polysaccharide with nontoxic and biodegradable advantages, obtained from the partial deacetylation of biopolymer chitin [11,12,13]. It is extensively used in the preparation of nanoparticles because of its ability to protect itself from degradation in the gastrointestinal tract by proteolytic enzymes [14]. However, the potential application of chitosan is limited by its low solubility in aqueous media, because the solubility in aqueous solutions above pH 7 is very weak, and it need to be dissolved in acidic aqueous solutions [15]. Carboxymethyl chitosan (CMCS) is a key derivative of modified chitosan. Their water solubility is better than chitosan, and it has the characteristics of moisturizing, film-forming and nontoxicity [16]. Thus, CMCS is a potential source for nanoparticle fabrication. Wu et al. determined the oxidative stability of krill oil-in-water emulsions without the use of chitosan or CMCS at various conditions [10]. Sheng et al. investigated the influences of the addition of fish gelatin or anionic heat-treated milk protein-based species on the oxidative stability of krill oil-in-water emulsions [17]. Haider et al. studied the fabrication, characterization, and oxidative stability of chitosan–tripolyphosphate nanoparticles using a two-step process [18]. However, to our knowledge, no information about CMCS nanoparticles as carriers for krill oil is available. This study focuses on the physical properties, oxidative stability and controlled release performances of CMCS nanoparticles loaded with krill oil. Firstly, RSM was applied in order to optimize the suitable extraction process of krill oil. The physical properties of the nanoparticles were characterized by a series of measurements, including particle size and surface charge (zeta potential), polydispersity index (PDI), encapsulated ratio and transmission electron microscopy (TEM). The oxidative stability of nanoparticles was evaluated by the peroxide value (POV), thiobarbituric acid-reactive substances (TBARS), pH value, and turbidity. Finally, the delivery performances of the nanoliposomes were determined by the release profiles of the krill oil in a simulated gastrointestinal environment.

## 2. Result and Discussion

### 2.1. Optimization of Extraction Parameters

#### 2.1.1. Predicted Model and Statistical Analysis

The influences of various proteases, such as neutral protease, trypsin, compound protease, bromelain and papain on the extraction yields of krill oil were primarily investigated. The results indicated that the recovery of the total lipids was highest in the utilization of the compound protease. In addition, several factors, including the enzyme concentration, enzymolysis time and enzymolysis temperature on the extraction yield were studied. According to single-factor tests, the factors of enzyme concentration (0.1%, 0.15%, and 0.2%), enzymolysis time (2, 3, and 4 h) and enzymolysis temperature (40, 45 and 50 °C), in order to achieve the optimal extraction condition were considered in the RSM design. The data were analyzed by Design-Expert software. As a result, the correlation between the lipid extraction yield and the variables was given as follows:

Y = 86.33 + 0.77X_1_ − 0.63X_2_ − 3.44X_3_ + 0.53X_1_ X_2_ + 0.19X_1_ X_3_ − 1.02X_2_ X_3_ − 3.07X_1_^2^ − 1.95X_2_^2^ − 13.07X_3_^2^ (1)

where Y is the extraction yield of krill oil, and X_1_, X_2_, and X_3_ are the enzyme concentration, enzymolysis time, and enzymolysis temperature, respectively.

As shown in Table 1, the *p*-value (<0.0001) indicated that the model was significant. The total determination coefficient (R_2_ = 0.9859) was satisfactory to validate the significance of the model. The linear terms (X_3_) and quadratic terms (X12, X22, and X32) presented significant effects on the extraction yields (*p* < 0.05). The results indicated that the enzymolysis temperature was the most significant parameter affecting the extraction yield of the lipids. Furthermore, the lack-of-fit was not significant relative to the pure error, which indicated that the experimental design could determine the effects of the independent variables on the extraction yields of the lipids. The coefficient of variation (CV) represents the reproducibility of the models. A previous report showed that a model can be considered to be reasonably reproducible if coefficient of variation (CV) is less than 10% [19]. Thus, the CV with 1.76% demonstrated the good reproducibility of the model in this study.

#### 2.1.2. Response Surface Plot

**Figure 1:**
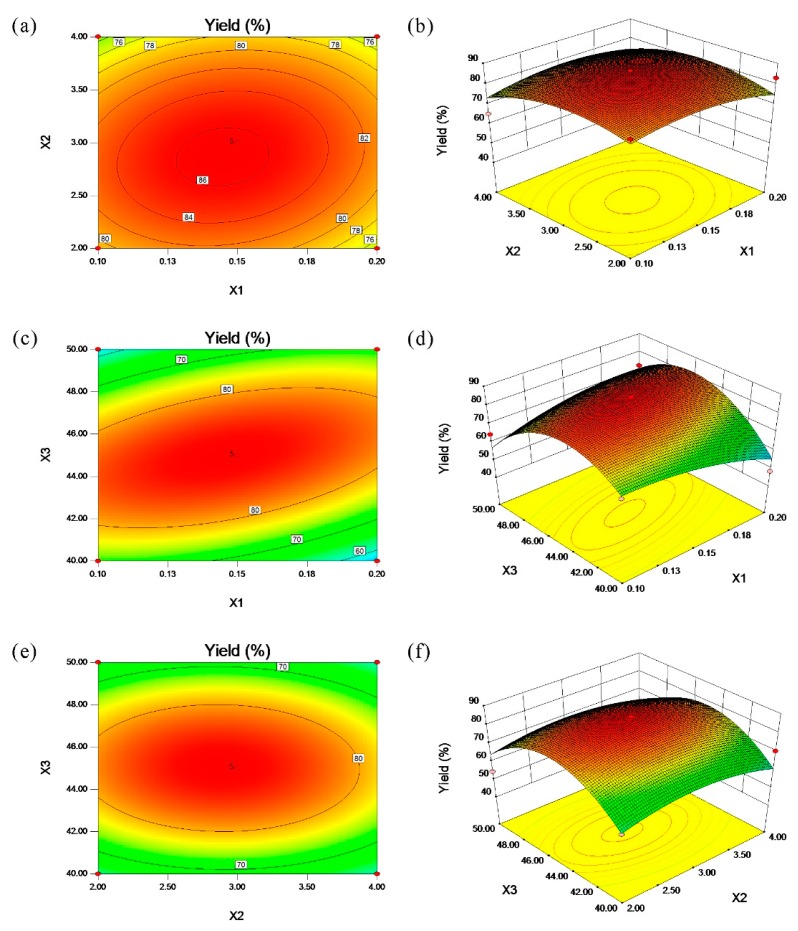
Contour plots (**a**,**c**,**e**) and response surface plots (**b**,**d**,**f**) showing the effects of the variables (X_1_, enzyme concentration; X_2_, enzymolysis time; and X_3_, enzymolysis temperature) and their mutual effects on the extraction yield of the krill oil.

The 3D surface plot and 2D contour plot are perfect ways to describe the interaction between the independent variables [20]. Figure 1a,b shows the influences of the enzyme concentration and enzymolysis time on the extraction yields of krill oil. It indicates that the extraction yield increased with the increase of the enzyme concentration and enzymolysis time, however, a slightly decrease happened when the extraction yield reached the highest level. A similar phenomenon between the enzymolysis temperature and enzyme concentration or enzymolysis time was shown in Figure 1c–f. Furthermore, all of the independent variables exhibited quadratic effects on the lipid recovery. In summary, the moderate enzyme concentration, enzymolysis time and temperature affected the extraction yield of the krill oil.

#### 2.1.3. Verification of Predictive Model

The predicted yield of the krill oil was 86.63% under the extraction conditions of an enzyme concentration of 0.16%, enzymolysis time of 2.89 h, and enzymolysis temperature of 44.37 °C. For operation convenience, the optimal parameters were modified as follows: enzyme concentration 0.16%, enzymolysis time 2.9 h and enzymolysis temperature 45 °C. The actual laboratorial value of the extraction yield of the krill oil was 86.02%. There were no significant differences between the predicted and actual values for the krill oil extraction. Consequently, the optimal conditions for the lipid extraction given by the RSM model was practical.

### 2.2. Separation of Phospholipid Classes

**Figure 2:**
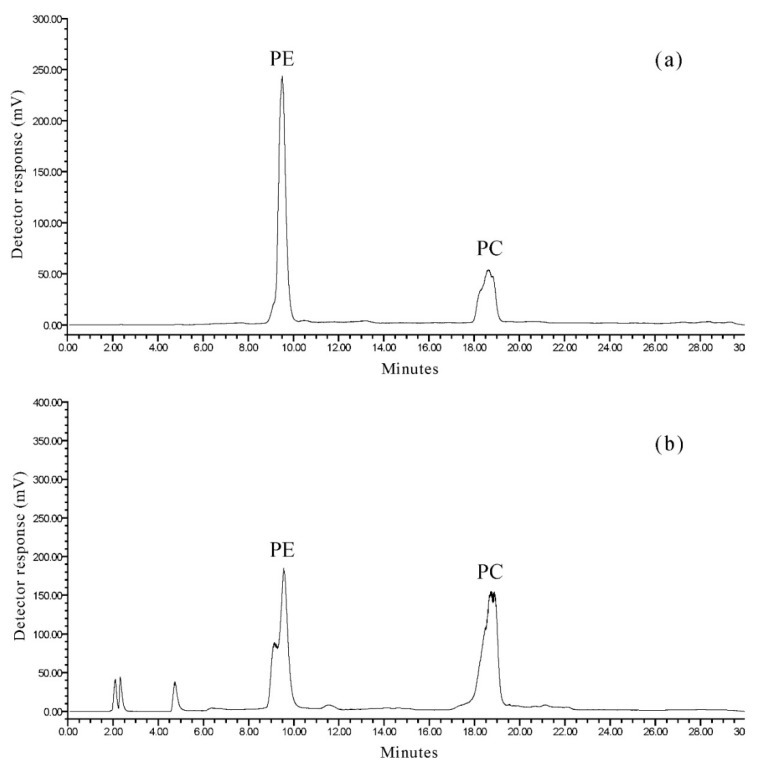
HPLC with an evaporative light-scattering detector (HPLC-ELSD) chromatograms of phospholipid classes identified in different samples. (**a**) Standard mixture; (**b**) *E. Superba.* Separation was performed using a thermo hypersil silica (150 × 4.6 mm, 3 µm) and a linear gradient ranging from n-hexane/isopropanol (3:2, *v/v*) to water, at a flow rate of 0.5 mL/min. The detection was performed using an ELS detector.

The phospholipid classes of the krill oil extracted from *Euphausia superba* were separated by HPLC with an evaporative light-scattering detector (HPLC-ELSD). Figure 2a shows the normal-phase HPLC chromatogram of the phospholipid standard mixtures (phosphatidylethanolamine (PE) and phosphatidylcholine (PC)). The PE and PC were separated well. Figure 2b exhibits the normal-phase HPLC chromatogram of the phospholipids in krill oil. Only two phospholipid classes, PE and PC were detected in *Euphausia superba* (Figure 2b). The quantification of phospholipid classes was based on external calibration. The ELSD response varies with the scattering domain and for a large range of sample sizes, the peak area (A) is usually related to the sample mass (m) by the equation (A = am*^b^*). In the equation, a and b have relations with size, shape, concentration, nature, number, and the speed of particles formed during the nebulization process [21]. Herein, the correlation between the experimental and calculated data were obtained using nitrogen at 45 °C. The coefficients a, b, and c of a quadratic model, (*y* = a*x*^2^ + b*x* + c), are given in Table 2 for each phospholipid class. The content of PE and PC in krill oil were 23.5 and 49.4 mg/g, respectively, of the total lipids. The results indicated that PC was predominant in the phospholipids of krill oil. The total phospholipid was 72.9 mg/g. It demonstrated that krill oil contained a high concentration of phospholipids.

### 2.3. Characterization of Nanoliposomes

According to the literature, liposomes with a particle size <200 nm can be defined as nanoliposomes. Table 3 shows that the particle sizes of liposomes exceeded 200 nm when the concentrations of CMCS were 0.3% and 0.5%, respectively. However, there was no significant difference in the encapsulation efficiency of nanoliposomes with the increase of CMCS concentration. The reason might be attributed to the amphiphilic property of CMCS, which does not destroy the structure of nanoliposomes. In addition, PDI is an evaluation index of particle size distribution in colloidal systems. The smaller the PDI, the narrower and more uniform the particle size distribution are. As shown in Table 3, the PDI values of all of the samples were 0.14–0.19, which reflected that the liposomes prepared in this experiment had a narrow particle size distribution. When the concentrations of CMCS were 0.1% and 0.4%, the encapsulated ratios were higher, 87.33% and 88.23%, respectively, and the particle sizes were small. The zeta potential provides some indirect information about the changes in the interfacial properties. CMCS is negatively charged, resulting in a negative charge of nanoliposomes. The zeta potentials of the nanoliposomes increased slightly with the increase of the concentration of CMCS, which indicated that CMCS was bound to the surface of the nanoliposomes, and the changes of the CMCS showed no significant increase in the zeta potential. According to a previous study [22], chitosan with a positive charge can be bonded with the nanoliposomes’ surfaces without charge or with a slightly negative charge. Although the electrostatic force between chitosan and liposomes is very weak, chitosan could bend to adapt to the liposomes with a larger curvature so as to achieve stability in the system. Hence, there is speculation that CMCS, as a linear polysaccharide with a negative charge, might be linked to the surface of the nanoliposomes in the same way.

**Figure 3:**
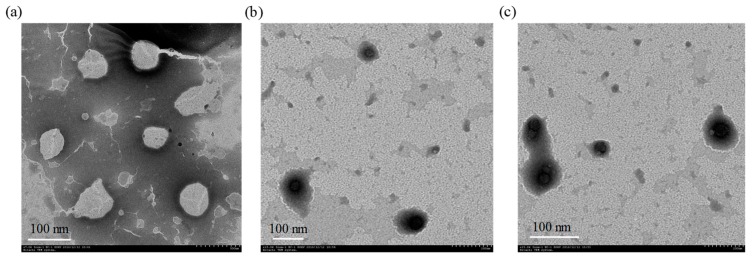
TEM micrograph of krill oil nanoliposomes with (**a**) 0.0% CMCS, (**b**) 0.1% CMCS, and (**c**) 0.4% CMCS. Scale bar represents 100 nm.

Additionally, the TEM studies showed that the krill oil nanoliposomes can be self-emulsified by phospholipids (Figure 3). The krill oil nanoliposomes are spherical in shape, with a 60 nm particle size. CMCS nanoliposomes are also spherical in shape, but the particle sizes are around 100 nm. which is not consistent with the dynamic light scattering (DLS) result. In other words, the diameters of the droplets measured by TEM were obviously smaller than those measured by DLS. Similar results were also reported by Roohinejad et al. [23]. The reason may be attributed to the difference in sample preparation. The liposomes measured by DLS are in a liquid state where the particles are relatively hydrated and swollen, while TEM involves a drying step that results in a micelle dehydration state and shrinkage [24]. In summary, the samples with a concentration of 0.1% CMCS and 0.4% CMCS were selected for further study, and the traditional nanoliposome without CMCS coating was used as a control sample.

### 2.4. Oxidative Stability of Nanoliposomes

**Figure 4:**
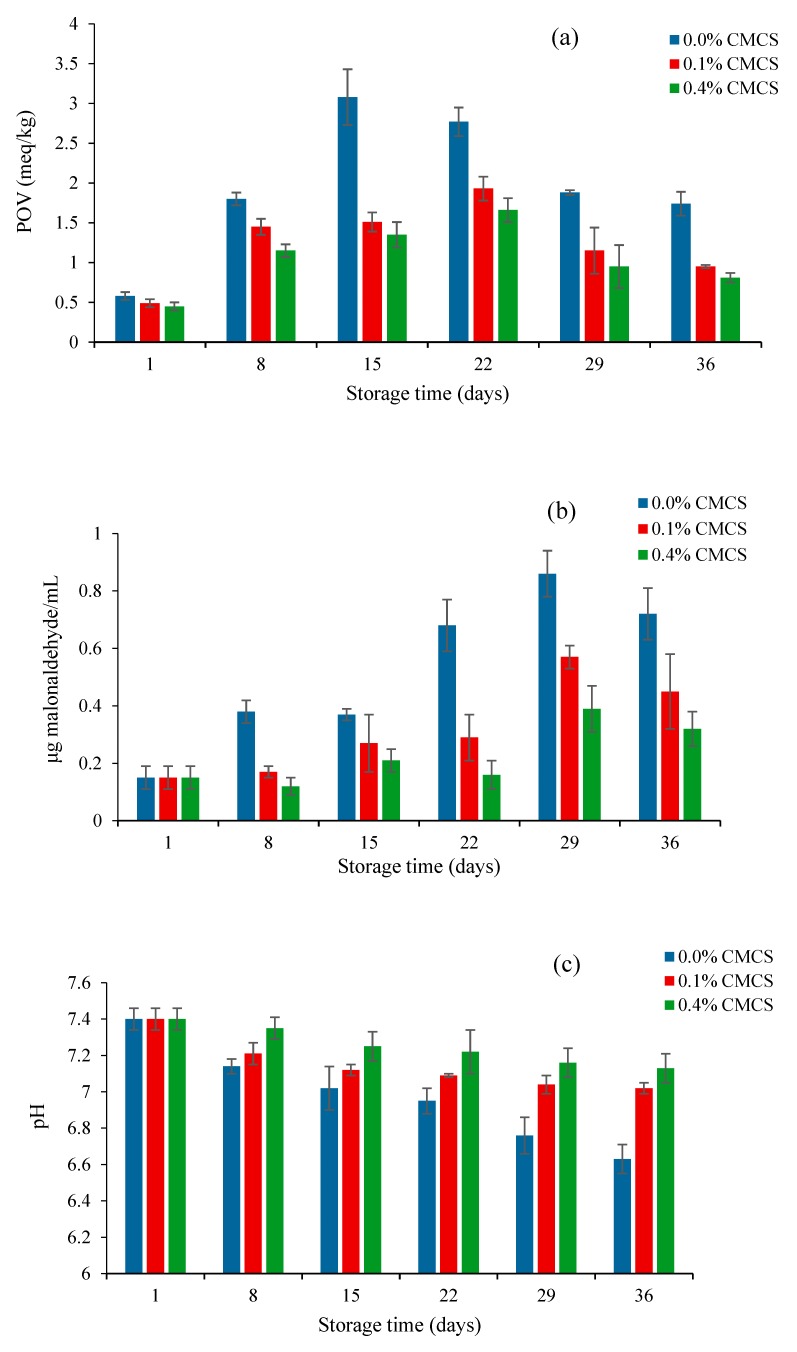

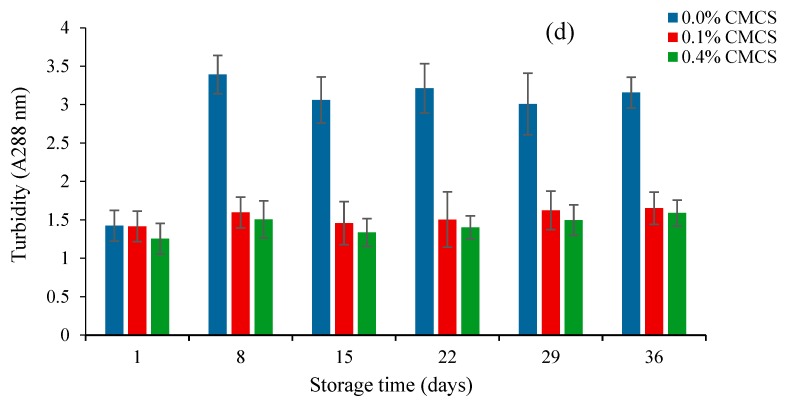
(**a**) Peroxide value (POV), (**b**) thiobarbituric acid-reactive substances (TBARS), (**c**) pH, and (**d**) turbidity as a function of time for krill oil nanoliposomes during storage at 4 °C. Values are means±standard deviation (SD) for at least three experiments.

The increase in the POV of the samples inflected the increasing formation of hydroperoxide, a primary lipid oxidation product. Lipid peroxidation is a chain reaction initiated by the hydrogen abstraction or the addition of an oxygen radical, caused by the oxidative decomposition of polyunsaturated fatty acids [25]. The nanoliposome without a CMCS coating showed a higher POV than the nanoliposomes with 0.1% CMCS and 0.4% CMCS, respectively. The increase of POV indicates that oxidation is more important than the decomposition of the oxidation products. With the prolongation of storage time, the decomposition was stronger than the oxidation. The reason might be due to the decomposition of hydroperoxides which led to the downward trend of POV in the three samples (Figure 4a).

TBARS values of nanoliposomes during 36 days of storage are given in Figure 4b. The increase in TBARS value indicated the formation of secondary lipid oxidation products, especially aldehydes [26]. Malondialdehyde is an important intermediate product of phospholipid oxidation. The measurement of TBARS value refers to the content of malondialdehyde in every mL of liposome solution. Therefore, the determination of the malondialdehyde content can indirectly reflect the degree of liposome oxidation. The malondialdehyde content in the three samples exhibited an upward trend before 29 days, and then decreased sharply. In addition, it is noteworthy that the content of malondialdehyde in CMCS nanoliposome was significantly lower compared to that observed in the empty CMCS nanoliposome. This indicated that the addition of CMCS can effectively prevent the excessive oxidation and improve the oxidative stability of krill oil liposome.

Lipids are liable to rancidity during storage, and the pH value can reflect the rancidity of the krill oil liposome. The lower the pH value, the higher the degree of rancidity. Figure 4c shows that the pH value of the empty CMCS liposome was obviously lower than the other samples. Moreover, the pH values of the CMCS nanoliposomes had no significant change, which also shows that CMCS can effectively prevent nanoliposomes from oxidation.

Turbidity is an effective indicator of the changes of the transparency of liposomes during storage. The higher the absorbance, the greater the turbidity. The breakdown and aggregation of liposomes would decrease the homogeneity of the solution and lead to an increase of absorbance. Figure 4d shows the variation in the turbidity of the nanoliposomes as a function of the storage time. The turbidities of the CMCS nanoliposomes had no significant change during storage, but for the empty CMCS coating liposome, the turbidity was obviously higher to that observed in the other two samples. The turbidity increased sharply in the first eight days, and then keep stable. It indicates that the CMCS coating could significantly improve the storage stability of the krill oil nanoliposome, and inhibit the increase of turbidity, which was caused by the leakage and aggregation of lipids. In addition, the particle sizes of the nanoliposomes had no significant changes with the increased of CMCS concentrations (Table 3). The turbidity of the nanoliposome (0.04% CMCS) showed a similar tendency to the nanoliposome (0.01% CMCS), indicating that the concentration of CMCS had no significant influence on the change of particle size.

### 2.5. In Vitro Release of Krill Oil from Nanoliposomes

**Figure 5:**
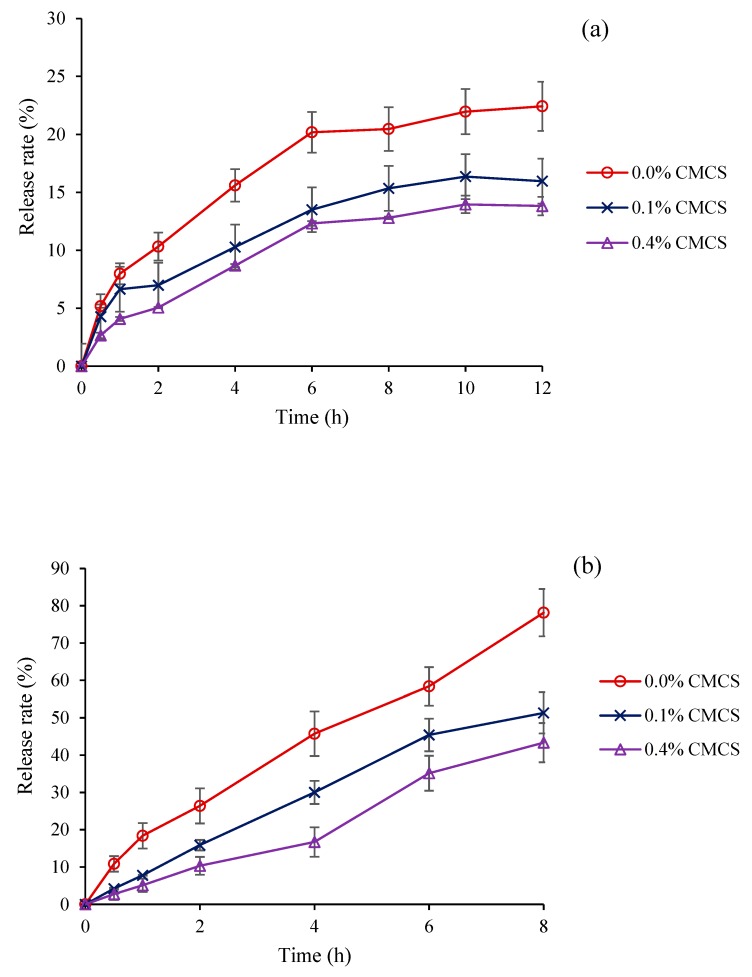
(**a**) Release rates of krill oil from nanoliposomes with different CMCS concentrations in a SGF environment; (**b**) release rates of krill oil from nanoliposomes at different CMCS concentrations in a SIF environment. Values are means ± SD for at least three experiments.

The role of a carrier system is to deliver functional elements to the desired target sites, promoting the exposure concentrations or duration per administered dose at the target sites. The small intestine is a key absorption site for functional components. However, before nanoliposomes reach the small intestine, they may swell or burst in stomach acid. CMCS is a widely used obstacle for loading functional compounds to the small intestine. The in vitro release of krill oil from nanoliposomes in a SGF environment is shown in Figure 5a. Compared with the nanoliposomes without a CMCS coating, the release rates of krill oil from the nanoliposomes with the CMCS coating were more controllable, with only 13.5% and 12.32% after 6 h. In a SIF environment, the CMCS nanoliposomes also exhibited preferably slow-releasing rates (Figure 5b). The release rate of an empty CMCS coating nanoliposome reached 78.15%, while it reached 43.35% and 51.34% for the CMCS nanoliposome after 8 h. Moreover, it is noteworthy that the release rates of the three nanoliposomes in the SGF environment were obviously lower than those in the SIF environment. The phenomenon demonstrated that the main site for the release of krill oil from nanoliposomes was in the small intestine, and the protective effect of CMCS in gastric juice was stronger than that in the intestinal juice. A similar phenomenon was also demonstrated by Sahoo et al. [27], the likely reason might be attributed to the unique pH sensitivity of CMCS. Because CMCS contains a large number of carboxyl groups and amino groups. In a SGF environment, ammonia acquires protons and the charge property of CMCS is determined by NH_3_^+^. The strong ionic bond binding force results in the difficulty of krill oil release from CMCS nanoliposomes. In contrast, the carboxyl group is largely dissociated, and exhibits a charged property of COO^−^ in the SIF environment, which makes krill oil more easily released from CMCS nanoliposomes. Therefore, CMCS nanoparticles as carriers for krill oil are available.

## 3. Materials and Methods

### 3.1. Materials and Chemicals

Frozen *Euphausia superba* meat was purchased from Dalian Marine Co., Ltd. (Dalian, China) and stored in −80 °C until use. Neutral protease, trypsin, compound protease, bromelain and papain were purchased from Nanjing sode Biotechnology Co., Ltd. (Jiangsu, China). Methanol, chloroform, trichloroacetic acid, thiobarbituric acid, pepsin, and bile salt were bought from Macklin chemical Reagent Co., Ltd. (Shanghai, China).

### 3.2. Krill Oil Extraction

The meat of *Euphausia superba* (100 g) was smashed and mixed well with distilled water (100 mL). Then, the pH value was adjusted to 6.5 with hydrochloric acid or sodium hydroxide (2 mol/L) by a pH meter. Several proteases (neutral protease, trypsin, compound protease, bromelain, and papain) with different concentrations (0.1%, 0.15% and 0.2%) were separately mixed with the samples and incubated in an orbital shaking bath with different temperatures. After hydrolysis, 50 mL of hexane/ethanol (7:3, *v/v*) was added and shaken mechanically for 40 min. Then, the suspensions were removed and the substrate were re-extracted twice with hexane (10 mL). The combined organic layers were evaporated by a rotary evaporator and further dried with pure nitrogen (99.99%). The total lipids were stored in −20 °C. For the determination of the total lipid content, the *Euphausia superba* meat was lyophilized and the total lipid was extracted by two extraction cycles using pressurized liquid extraction methodology according to our previous study [6].

The extraction yield (%) was expressed as follows:

$$\mathrm{Extraction}\;\mathrm{yield}\;\left(\%\right)=\frac{m_{{}^{\circ}}}{m}\times 100$$

where $m_{\circ }$ is the lipid content (g) extracted by enzymatic hydrolysis and *m* is the total lipid content of lyophilized krill powder (g) extracted by pressurized liquid extraction.

### 3.3. Experimental Design of RSM

The effects of the enzyme types, enzyme concentration, enzymatic hydrolysis time and temperature on the extraction yields of krill oil were investigated by a single-factor test (data not shown). According to the results, three primary factors, namely the enzyme concentration, enzymatic hydrolysis time and temperature, were chosen. The optimum combination of variables, including the enzyme concentration (*X*_1_), enzymatic hydrolysis time (*X*_2_), and temperature (*X*_3_) were determined by the Box−Behnken design (BBD). The three factors were coded at three levels (−1, 0, 1, respectively, as shown in Table 4) in the extraction process.

The variables were coded according to the following equation:

Xi=(χi−χ0)Δχi

where *X_i_* is the coded value of independent variable, *χ_i_* is the actual value of the independent variable, *χ*_0_ is the real value of the independent variable at the center point, and Δχ*_i_* is the step change of the variable.

The whole design was composed of 17 tests. Of these, 12 tests were factorial points and 5 tests were axial points. The experiments were carried out in second-order polynomial mode, as follows:

$$Y=\overset{3}{\underset{i=1}{\sum }}\beta_{i}X_{i}+\overset{3}{\underset{i=1}{\sum }}\beta_{ii}X_{i}^{2}+\overset{2}{\underset{i=1}{\sum }}\overset{3}{\underset{j=i+1}{\sum }}\beta_{ij}X_{i}X_{j}$$

where *Y*, the lipid extraction yield of *Euphausia superba*, is the predicted response; *β*_0_, *β_ij_* and *β_ij_* are the mean regression coefficients for the intercept, linear, quadratic and interaction terms, respectively; and *X_i_* and *X*_j_ are the independent variables (*i* ≠ *j*).

### 3.4. Separation of Phospholipid Classes of Krill Oil

The phospholipid profiles of krill oil were determined in an Agilent 1100 HPLC system equipped with a 2424 ELS detector. The drift tube temperature was set at 45 °C. The phospholipid classes were separated with the use of thermo hypersil silica (150 × 4.6 mm, 3 µm) with a flow rate of 0.5 mL/min. The separations were performed with a 26 min gradient elution ranging from n-hexane/isopropanol (3:2, *v/v*) to water. 

### 3.5. Preparation of Nanoliposome

The nanoliposome was prepared by homogenizing 1 wt% krill oil and 99 wt% 10 mM acetic acid buffer (pH 7) using an Ultra-Turrax T18 homogenizer operating at 19,000 rpm for 2 min. It was further homogenized by passing it twice through a high-pressure homogenizer (D-3L, PhD Tech., Newark, DE, USA) at 800 bar for 3 min. Sodium azide (10%) was added to the emulsions to inhibit microbial growth. Chitosan was dissolved in a 10 mM acetic acid buffer. Then, the prepared nanoliposomes were dropped into a series of different concentrations of chitosan solutions (1:1, *v/v*) using a magnetic stirrer. The dripping speed was 1 drop/s. After the mixing process, the solutions continued to be stirred for 2 h at 300 r/min. The chitosan nanoliposomes were finally prepared, then stored in darkness at 4 °C for 36 days. The samples were taken on day 0, 8, 15, 22, 29, and 36, and were stored at −20 °C until the oxidative stability analysis.

### 3.6. Characterization of Nanoliposomes

The size of the initial nanoliposome droplets was observed by an TE2000-S fluorescent inverted microscope after the sample was dropped on glass slides. The morphologies of the nanoliposomes were imaged using a transmission electron microscopy (TEM) machine (JEOL H7650, Hitachi High-Technologies Corp., Tokyo, Japan) The nanoliposome was diluted 10 times with a buffer, and then one drop of the suspension was placed on a copper grid. The grid was air-dried before being transferred into the microscope. The images of the nanoliposomes were recorded.

The particle size, polydispersity index (PDI) and zeta potential of the nanoliposomes were measured by dynamic light scattering instruments (Zetasizer Nano-ZS, Malvern Instruments, Malvern, UK). All of the samples were diluted with deionized water (1:100) before measurement in order to avoid multiple scattering effects. A particle refractive index of 1.469 and a dispersant refractive index of 1.330 (water) were used for all of the nanoliposome samples. All of the samples were measured in duplicate. 

### 3.7. Encapsulated Ratio of Krill Oil

Firstly, krill oil was dissolved in n-hexane (1:15, *v/v*) as a sample, and the absorbance at 200–400 nm was measured by a UV/VIS spectrophotometer with a slight modification [28]. The n-hexane was used as blank reference. The results showed that the nanoliposome at 217 nm had a strong absorption. Consequently, 217 nm was chosen for the following step.

Then, 10 mL of the nanoliposome sample was centrifuged at 4000 rpm for 8 min at 4 °C. Then, 10 mL of n-hexane was added and was shaken mechanically for 8 min. The supernatant was separated and 10 mL of 5% triton-x 100 methanol as a de-emulsifier was added into the solution and underwent ultrasound for several minutes until the transparent solution was obtained. Finally, n-hexane was used to extract the krill oil. The absorbance at 217 nm was measured. The encapsulated ratio was calculated by the following equation:

$$\mathrm{Encapsulated}\;\mathrm{ratio}\;\left(\%\right)=\frac{\;\mathrm{Amount}\;\mathrm{of}\;\mathrm{encapsulated}\;\mathrm{krill}\;\mathrm{oil}\;}{\mathrm{Total}\;\mathrm{amount}\;\mathrm{of}\;\mathrm{added}\;\mathrm{krill}\;\mathrm{oil}}$$

### 3.8. Measurements of Lipid Oxidation

#### 3.8.1. Peroxide Value (POV)

The POV was measured according to the reported method [29] with a slight modification. Then, 10 mL of the nanoliposome sample was dissolved in 10 mL of CHCl_3_:CH_3_OH (7:3, *v/v*) by vortexing (for 20 s, three times) and then isolated the organic solvent phase by centrifugation at 4000× *g* for 3 min. The organic layer (3 mL) was suspended in CHCl_3_:CH_3_OH (7:3, *v/v*), followed by 50 μL of ammonium thiocyanate and 50 μL of a ferrous chloride solution. The absorbance of the solution was measured at 500 nm using a V-1200 UV/VIS spectrophotometer. The POV was expressed as meq/kg sample.

#### 3.8.2. Thiobarbituric Acid-Reactive Substances (TBARS)

The TBARS were measured according to a previous study [28]. Then, 4 mL of a nanoliposome sample and 10 mL of a TBA reagent (15% *w/v* trichloroacetic acid, 0.375% *w/v* thiobarbituric acid and 0.25 M HCl) were vortexed in glass test tubes. The tubes were incubated at 90 °C for 30 min and cooled to room temperature, then centrifuged (4000× *g*) for 10 min. The absorbance of the supernatant was measured at 532 nm and the results were expressed as μg malonaldehyde/mL nanoliposome.

#### 3.8.3. pH Value

The pH values of the nanoliposomes were measured using a pH meter (FiveEasy Plus, Mettler-Toledo, Columbus, OH, USA). All of the samples were determined in triplicate.

#### 3.8.4. Turbidity 

The turbidities of the samples were measured by the previous description [30]. Then, 1 mL of nanoliposome was diluted to 10 mL with an acetic acid buffer (pH 7). The turbidity was measured at 288 nm using a TU-1900 UV/Visible spectrophotometer. All of the samples were withdrawn periodically for the turbidity measurements in triplicate.

### 3.9. In Vitro Release of Krill Oil from Nanoliposomes

The in vitro release profiles of krill oil from nanoliposomes were analyzed using the dialysis method. The simulated gastric fluid (SGF) consisted of 0.1 N HCl (pH 1.2) with 0.1% pepsin. The simulated intestinal fluid (SIF) solution (pH 7.4) was a phosphate buffer saline (PBS; pH 7.4) with 0.2 mg/L of bile salt. The nanoliposomes (40 mL) were dispersed in 100 mL of a SGF solution or SIF solution, then immediately loaded into dialysis bags. Afterwards, the dialysis bags were placed in an acid release medium (0.1 N HCl, pH 1.2, and PBS 7.4) at 37 °C. Aliquots of the dissolution medium (10 mL) were withdrawn, and the same volume of buffer (10 mL) was fed back to the release medium. The percentage cumulative quantity of krill oil released from the nanoliposomes was determined as a function of time.

### 3.10. Statistical Analysis

The data were exhibited as the mean ± SD from three replicates for each assay. Statistical significance was analyzed by one-way ANOVA with SPSS 16.0 software (SPSS Inc., Chicago, IL, USA). The Student’s *t*-test was used to determine the significant differences. The significance level was considered to be *p* < 0.05.

## 4. Conclusions

In this study, the optimal conditions for krill oil extraction were as follows: enzyme concentration of 0.16%, enzymolysis time of 2.9 h, and enzymolysis temperature of 45 °C. Under these conditions, the extraction yield can reach 86.02%. The oxidative stability of CMCS nanoliposomes was investigated by five assays. The results show that CMCS nanoliposomes can obviously decrease the POV and TBARS values, inhibit the decrease of the pH value and the increase of turbidity. Furthermore, the oxidative stability of the nanoliposomes increase with the increase of the CMCS concentration. It indicated that the CMCS nanoliposome can effectively enhance the oxidative stability of krill oil during storage. Accordingly, the in vitro release profiles showed that CMCS nanoliposomes could control the release of krill oil in a simulated gastrointestinal environment. Therefore, CMCS nanoliposomes can be used as potential oral delivery systems for functional oil.5. 

## Figures and Tables

**Table 1:** Analysis of variance (ANOVA) for the response surface quadratic model.

Source	Df *^d^*	Mean Square	*F*-Value	*p*-Value
Model	9	102.34	54.46	<0.0001
*X*_1_*^a^*	1	4.71	2.51	0.1573
*X*_2_*^b^*	1	3.21	1.71	0.2323
*X*_3_*^c^*	1	94.60	50.34	0.0002
*X*_1_*X*_2_	1	1.13	0.60	0.4627
*X*_1_*X*_3_	1	0.14	0.075	0.7923
*X*_2_*X*_3_	1	4.12	2.19	0.1822
*X*_1_^2^	1	39.75	21.15	0.0025
*X*_2_^2^	1	15.93	8.48	0.0226
*X*_3_^2^	1	719.26	382.74	<0.0001
Lack of fit	3	13.15	4.38	
Pure error	4	0.000	0.000	
R^2^			0.9859	
CV%			1.76%	

*^a^ X*_1_—enzyme concentration; *^b^ X*_2_—enzymolysis time; *^c^ X*_3_—enzymolysis temperature; *^d^* df—degrees of freedom; CV—coefficient of variation.

**Table 2:** Coefficients a, b, and c of the quadratic standard curves obtained for each phospholipid class.

PL Class	Equation: a*x*^2^ + b*x* + c			*R^2^*
	*a*	*b*	*c*	
PE	6.0 × 10^7^	1.0 × 10^7^	155,617	0.9991
PC	5.0 × 10^7^	1.0 × 10^7^	40,616	0.9998

**Table 3:** Effect of the concentration of carboxymethyl chitosan (CMCS) on the physical properties of nanoliposomes. PDI—polydispersity index.

CMCS (*w/v*)	Particle Size (nm)	PDI	Zeta Potential	Encapsulated Ratio (%)
0.0%	185.07 ± 2.15	0.14 ± 0.03	−38.43 ± 0.11	85.4 ± 1.4
0.1%	181.56 ± 3.16	0.16 ± 0.02	−38.84 ± 1.36	87.33 ± 0.92
0.2%	194.3 ± 2.2	0.19 ± 0.02	−40.26 ± 0.82	84.6 ± 0.2
0.3%	202.73 ± 3.14	0.15 ± 0.00	−41.67 ± 0.47	84.79 ± 1.58
0.4%	185.67 ± 2.45	0.15 ± 0.03	−42.18 ± 0.92	88.23 ± 1.27
0.5%	228.16 ± 4.19	0.19 ± 0.01	−42.09 ± 1.39	86.45 ± 2.04

**Table 4:** Experimental and predicted values for the lipid yield by the Box-Behnken design.

Run	Uncoded Variables			Coded Variables *^a^*			Lipid Yield (*Y*), %	
	χ_1_	χ_2_	χ_3_	*X*_1_	*X*_2_	*X*_3_	Experimental	Predicted
1	0.15	3	45	0	0	0	86.33	86.33
2	0.10	2	45	−1	−1	0	81.33	81.71
3	0.15	4	40	0	1	−1	76.46	75.13
4	0.20	2	45	1	−1	0	82.96	82.18
5	0.15	3	45	0	0	0	86.33	86.33
6	0.15	2	50	0	−1	1	68.2	69.52
7	0.10	4	45	−1	1	0	78.6	79.38
8	0.10	3	50	−1	0	1	67.5	65.79
9	0.10	3	40	−1	0	−1	72.5	73.05
10	0.15	3	45	0	0	0	86.33	86.33
11	0.15	3	45	0	0	0	86.33	86.33
12	0.20	4	45	1	1	0	82.36	81.98
13	0.15	2	40	0	−1	−1	75.3	74.37
14	0.20	3	40	1	0	−1	72.5	74.21
15	0.15	3	45	0	0	0	86.33	86.33
16	0.15	4	50	0	1	1	65.3	66.23
17	0.20	3	50	1	0	1	68.25	67.70

*^a^ X*_1_—enzyme concentration; *X*_2_—enzymolysis time; *X*_3_—enzymolysis temperature.
